# Isolation, Screening, and Identification of Novel Isolates of Actinomycetes from India for Antimicrobial Applications

**DOI:** 10.3389/fmicb.2016.01921

**Published:** 2016-12-06

**Authors:** Vineeta Singh, Shafiul Haque, Harshita Singh, Jyoti Verma, Kumari Vibha, Rajbir Singh, Arshad Jawed, C. K. M. Tripathi

**Affiliations:** ^1^Microbiology Division, Council of Scientific and Industrial Research—Central Drug Research InstituteLucknow, India; ^2^Department of Biotechnology, Institute of Engineering and TechnologyLucknow, India; ^3^Department of Biosciences, Jamia Millia Islamia (A Central University)New Delhi, India; ^4^Research and Scientific Studies Unit, College of Nursing and Allied Health Sciences, Jazan UniversityJazan, Saudi Arabia; ^5^Fermentation Technology Division, Council of Scientific and Industrial Research—Central Drug Research InstituteLucknow, India; ^6^Department of Biotechnology, Himachal Pradesh UniversityShimla, India; ^7^Department of Biotechnology, Shri Ramswaroop Memorial UniversityLucknow, India

**Keywords:** antibiotic production, antimicrobial activity, *Streptomyces* sp., chromone antibiotics, non-polyenes

## Abstract

The search for novel bioactive compounds from the natural environment has rapidly been gaining momentum with the increase in multi-drug resistant (MDR) pathogens. In the present study, the antimicrobial potential of novel actinomycetes has been evaluated by initial screening of six soil samples. Primary and secondary screening was performed against *Bacillus subtilis, Staphylococcus aureus, Escherichia coli, Candida albicans, Candida tropicalis, Trichophyton rubrum*, and other MDR bacterial and fungal test strains, thirteen active isolates were selected for further study. Microbial strains were identified on the basis of growth conditions and other biochemical characters. Five most active microbial strains were identified using 16S rRNA sequence homology and designated as *Streptomyces xanthophaeus* MTCC 11938, *Streptomyces variabilis* MTCC 12266, *Streptomyces xanthochromogenes* MTCC 11937, *Streptomyces levis* EU 124569, and *Streptomyces* sp. NCIM 5500. Four antibacterial and three antifungal compounds isolated from the above five isolates were purified and partially characterized using UV absorption and IR spectra. Two antibacterial metabolites, belong to chromone and peptide antibiotic, respectively. The antifungal compounds were found to be of non-polyene nature. In conclusion, we study the isolation of novel bacterial strains of actinomycetes for producing novel compounds having antibacterial and antifungal activities from the unexplored agro-ecological niches of India. Also, this study paves the way for further characterization of these isolates of *Streptomyces* sp. for their optimum utilization for antimicrobial purposes.

## Introduction

The search for bioactive metabolites including novel antibiotic compounds from microbial sources for potential use in agricultural, pharmaceutical, and industrial applications has become more important due to the development of drug/multi-drug resistance in most of the pathogenic microbes. Researchers across the globe are aggressively searching for new, potent, sustainable, and broad-spectrum antimicrobial compounds from various sources including microbes (Berdy, 2005; Hayakawa, 2008; Praveen et al., 2008; Singh and Tripathi, 2011). In natural soil habitat, *Streptomyces* are usually a major proportion of the total actinomycetes population and recognized as prolific producers of useful bioactive compounds (Tanaka and Omura, 1990; Kekuda et al., 2014).

Traditional screening methods have led to the isolation of common microorganisms capable of producing metabolites, which have already been extensively studied and established (Okami and Hotta, 1988; Kurtböke et al., 1992). Among the current strategies of natural-product screening, improved methodologies for isolating the uncommon and less studied rare actinomycetes are required to avoid the repeated isolation of the strains that produce known bioactive metabolites, and to improve the quality of the screened natural products (Takahashi and Omura, 2003; Berdy, 2005; Singh et al., 2009).

Likewise, the traditional methods of species classification and the identification of the organism(s) are mainly based on morphological, physiological, biochemical, developmental, and nutritional characters, and it is not adequate, and warrants for the use of molecular level approaches for assigning accurate taxonomic classification. Hence, precise assignment of taxonomic status to the novel bioactive microbial isolates through existing predictive bioinformatics methods and tools are very essential and aid in chemical characterization of the active molecules.

India has a unique asset of biodiversity, which can be used as a treasure for the search of novel isolates. With the variation of type of soil, according to the geographical changes, soil provide very complex habitat to the microbes residing in it. Due to this intricate environment, the soil microbes play an important role in the isolation of novel drugs. Among soil microbes, the members of *Streptomyces* sp. of actinomycetes, have been widely exploited for the production of commercially important secondary metabolites and enzymes. In the present study, the soil samples from six different unexplored agro-ecological niches of India have been screened-out to isolate *Streptomyces* sp. possessing antibacterial and antifungal activities.

## Materials and methods

### Collection of soil samples

The soil samples were collected from six diverse habitats of India (Lucknow, Uttar Pradesh: 26.7°N, 80.9°E; Badrinath, Uttarakhand: 30.7440°N, 79.4930°E; Delhi, New Delhi: 28.6100°N, 77.2300°E; Bhatinda, Punjab: 30.2300° N, 74.9519° E; Haryana: 28.04°N 76.11°E; Thinmala range, Kerala: 8.5074°N, 76.9730°E) for the isolation of microbes. These habitats included rhizosphere of the plants, agricultural soil, hospital surroundings, river mud, and preserved areas of forest soils. The samples were collected from upto 20 cm depth after the removal of ~3.0 cm of the soil from the surface. The soil samples were collected in polyethylene bags, sealed, and stored in a refrigerator. All chemicals, media, media components, and other reagents were purchased from Sigma-Aldrich (USA), Merck (USA), HiMedia Laboratories (India) etc.

### Pre-treatment of soil samples and isolation of cultures

The soil samples collected from different geographical areas were pre-treated to eliminate the commonly found microbes using physico-chemical methods. For the physico-chemical treatment of the soil samples, one gram of each soil sample was suspended in 10 ml of normal saline and distributed in aliquots. One aliquot of the soil sample was treated with heat for 1 h at 120°C and the other was treated with 1.5% phenol for 30 min at 30°C as described by Hayakawa et al. (1991). Afterwards, the physico-chemically treated soil samples were vortexed and left for 30 min, there after soil samples were serially diluted and 100 μl of each “dilution” was plated on nutrient agar (NA), actinomycetes isolation agar (AIA), yeast malt glucose agar (M6), antibiotic assay agar, starch casein agar, and Czapek Dox agar.

### Screening of microbial cultures

Primary screening for evaluating the antimicrobial potential of the axenic cultures was performed by perpendicular streak method of Madigan et al. (1997) against the bacterial strains of *Bacillus subtilis* MTCC 441, *Staphylococcus aureus* MTCC 96, *Escherichia coli* MTCC 64, and *Candida albicans* MTCC 183. Isolates were screened for antagonism studies by inoculating a single streak of the pure producer organism in the middle of the assay media plate. The plates were incubated for 4 days at 28°C and subsequently seeded with “test” organism by a single streak at a 90° angle to the streak of the “producer strain” and finally the plates were incubated for 1–2 days at 28°C. The microbial interactions were analyzed by determining the distance of inhibition measured in mm.

Microbial strains showing “moderate” to “good” inhibition activity were selected for secondary screening, which was performed by agar well method (Wu, 1984), using 100 μl of their fermented broth against *B. subtilis* MTCC 441, *B. subtilis* MTCC 121, *B. pumilus* MTCC 1607, *S. aureus* MTCC 902, *S. aureus* MTCC 96, *E. coli* MTCC 1304, *Salmonella typhi* MTCC 734, *Pseudomonas aeruginosa* MTCC 741, *Proteus vulgaris* MTCC 426, *C. albicans* MTCC 3017, *C. albicans* MTCC 183, *C. tropicalis* MTCC 184, *Saccharomyces cerevisiae* MTCC 170, *Cryptococcus terreus* MTCC 1716, *Aspergillus niger* MTCC 1344, *Trichophyton rubrum* MTCC 296, *Penicillium chrysogenum* MTCC 2725, and *Beauveria bassiana* MTCC 4564. All the experiments were performed in triplicate and the average values were considered for analysis.

### Characterization of microbial strain(s) from the selected cultures

The cultural characteristics of the producer strains were studied according to the method of Shirling and Gottlieb (1966) based upon their intensity of growth, growth pattern, colony color along with the color of aerial and substrate mycelia, and the formation of soluble pigments on oat meal agar (ISP-3), inorganic salt starch agar (ISP-4), glycerol asparagine agar (ISP-5), peptone yeast extract iron agar (ISP6), and tyrosine agar (ISP-7). The strains were characterized by streaking the culture(s) on the above mentioned medium plates and observed after 7–10 days of incubation at 28°C for the given characteristics.

Physiological and biochemical tests were performed as described by Williams et al. (1989) and Bergey's manual (Holt et al., 1994), and results were observed after 10 days of incubation of plates at 28°C. In addition, the strains were tested for nitrate reduction, tolerance to NaCl, decomposition of citrate, tartrate, acetate and pyruvate, and pH and temperature tolerance. The enzymatic activity assays of urease, amylase, protease and catalase were performed as suggested by Hopwood and Wright (1973). Finally, on the basis of macroscopic, biochemical and physiological characteristics hierarchical cluster analysis was performed by using PASW Statistics (formerly SPSS Statistics) Version 18 software program and dendrogram was generated based on the average linkage between the groups.

### Metabolite production

Pure and active cultures of microbial strains selected from the secondary screening experiments were grown in X-medium (g/l: Soybean meal, 10; CaCO_3_, 3; MgSO_4_.7H_2_O, 0.5; (NH_4_)_2_HPO_4_, 0.5; NaCl, 3; K_2_HPO_4_, 1; glycerol, 15 ml; DW 1, pH 6.9–7.0), and incubated at 28°C for 3–5 days and cellular growth was confirmed by visible pellets, clumps, aggregates or turbidity in the culture broth. The culture broths were centrifuged separately and filtrates were used to evaluate the antimicrobial activity against the above mentioned test microorganisms. Antibiotic activities of the strains were compared with that of known commercially available erythromycin (E^15^) and amphotericin B (AmB^100^).

### Extraction, purification, and partial characterization of the active compounds

Fermented culture was centrifuged at 10,000 rpm for 20 min to separate the biomass. The active metabolite was recovered from the fermented broth using two phase solvent extraction system with organic solvent. Solvents containing the active compounds were concentrated under vacuum to get “dried crude.” The obtained “crude” was treated with non-polar solvents like hexane or chloroform to separate the polar and non-polar components. The active components were purified by adsorption chromatography using silica gel (pore size 60 Å, mesh size: 230–400, particle size 40–63 μm) as a stationary phase and gel filtration chromatography using sephadex LH-20. The eluted fractions were assayed for their bioactivity against *B. subtilis* ATCC 6633 and *C. albicans* ATCC 24433 by disc diffusion method (Wu, 1984). The purity of the active fractions was further checked by high pressure liquid chromatography (HPLC) using reverse phase silica column (RP18). Finally, the UV spectra (Perkin Elmer Lambda-25 UV spectrophotometer) of various antibacterial and antifungal compounds were determined in methanol at 200–500 nm wave length.

## Results

### Screening of the active strains

**Figure 1:**
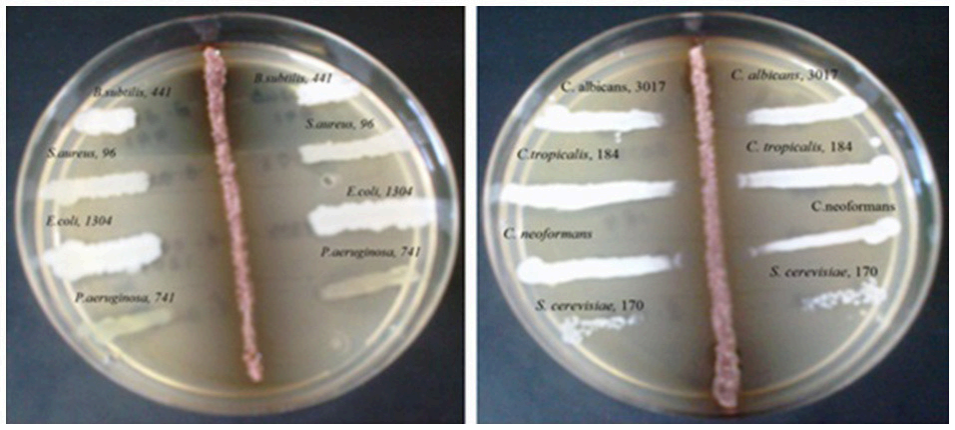
**Primary screening using perpendicular streak method for antibacterial and antifungal activity of the soil isolate (ZA25)**.

**Figure 2:**
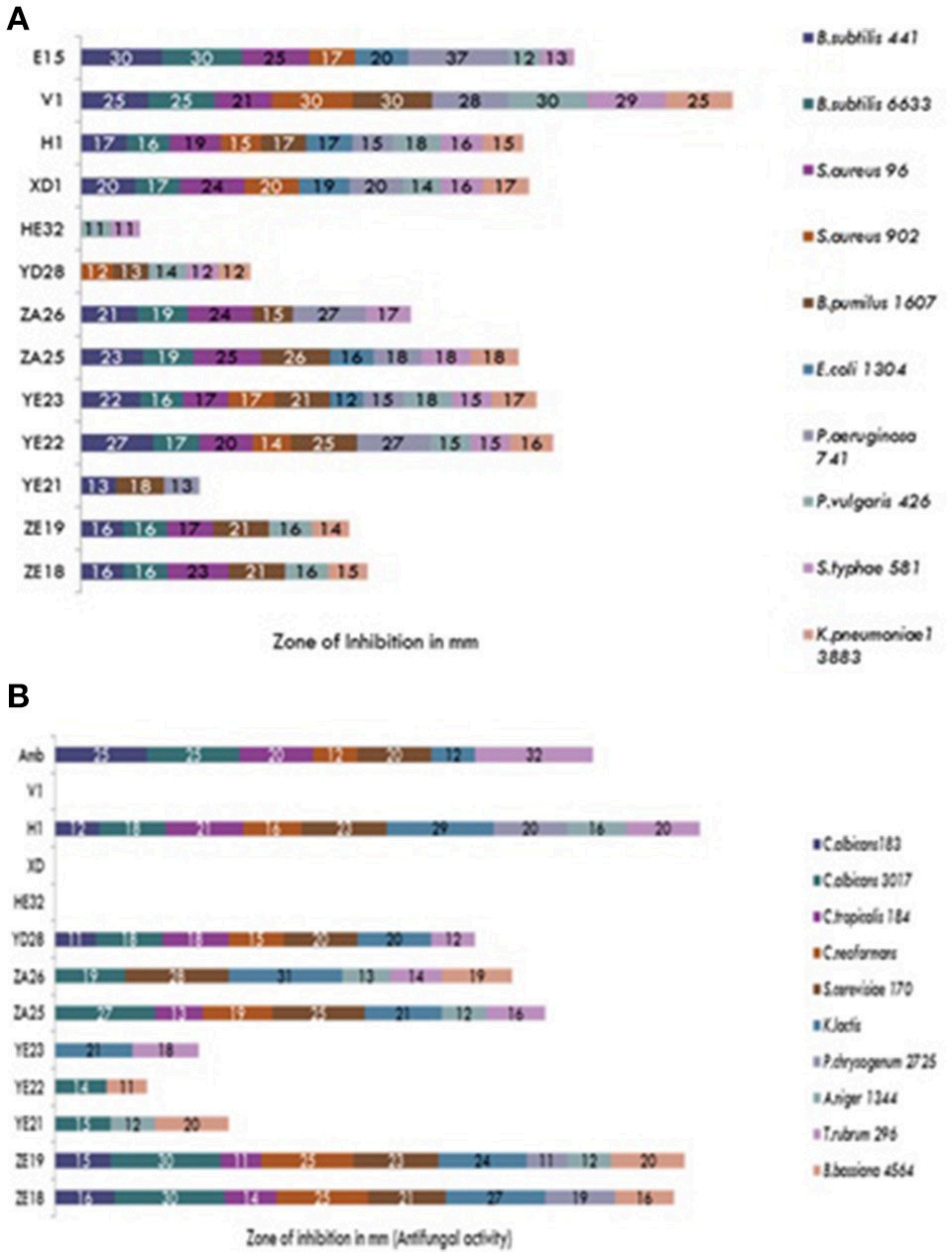
**(A)** Secondary screening of the soil isolates for the antibacterial activities. **(B)** Secondary screening of the soil isolates for the antifungal activities.

During the screening, thirty six actinomycetes were isolated from six different stressed agro-ecological niches of India. Microbial colonies showing distinct morphological characters were selected for the primary screening (Figure 1). Out of 36 total actinomycetes isolates, 15 showed moderate to strong antimicrobial activity against gram positive (*B. subtilis* MTCC6633, *S. aureus* MTCC 6538), gram negative (*E. coli* MTCC 1304) and fungal strain (*C. albicans* MTCC 1346). The active strains (actinomycetes) were further subjected to the secondary screening against some multi-drug resistant (MDR) bacterial as well as fungal test strains (Tables 1, 2). The screening results suggested that most of the isolates were active against gram positive bacteria in comparison with gram negative bacteria (Figures 2A,B). Out of 15 active isolates, 13 showed strong antimicrobial activity and were selected for detailed taxonomic, physiological, and biochemical studies.

**Table 1:** **Secondary screening (antibacterial activity)**.

**Sr. No**	**Test strains**	**Soil isolates (zone of Inhibition in mm)**															**PC**
		**ZE 18**	**ZE 19**	**YE 21**	**YE 22**	**YE 23**	**ZA 25**	**ZA 26**	**YD 28**	**RS 25**	**XD1**	**H1**	**V1**	**N1**	**S1**	**K1**	**E^15^**
1	*Bacillus subtilis* MTCC 441	16	16	13	27	22	23	21	–	–	20	17	25	12	13	11	30
2	*Bacillus subtilis* MTCC 121	16	16	–	17	16	19	19	–	–	17	16	25	–	12	–	30
3	*Bacillus pumilus* MTCC 1607	21	21	18	25	21	26	15	13	–	–	17	30	–	–	–	–
4	*Staphylococcus aureus* MTCC 96	23	17	–	20	17	25	24	–	24	24	20	21	–	13	11	25
5	*Staphylococcus aureus*, MTCC 902	–	–	–	14	17	–	–	12	25	20	15	30	–	14	11	17
6	*Escherichia coli* MTCC 1304	–	–	–	–	12	16	–	–	–	19	17	–	–	–	–	20
7	*Pseudomonas aeruginosa* MTCC 741	–	–	13	27	15	18	27	–	20	20	15	28		–	–	37
8	*Proteus vulgaris* MTCC 426	16	16	–	15	18	–	–	14	–	14	18	30	–	12	–	12
9	*Salmonella typhi* MTCC 581	–	–	–	15	15	18	17	12	–	16	16	29	–	–	–	13
10	*Klebsiella pneumonia* MTCC 13883	15	14	–	16	17	18	–	12	23	17	15	25	–	–	–	–

*(−), not active; PC, Positive Control; E^15^, Erythromycin and well diameter = 10 mm*.

**Table 2:** **Secondary screening (antifungal activity)**.

**Sr. No**	**Test strains**	**Soil isolates (zone of inhibition in mm)**															**PC**
		**ZE 18**	**ZE 19**	**YE 21**	**YE 22**	**YE 23**	**ZA 25**	**ZA 26**	**YD 28**	**RS 25**	**XD1**	**H1**	**V1**	**N1**	**S1**	**K1**	**AmB**
1.	*Candida albicans* MTCC 183	16	15	–	–	–	–	–	11	12	–	12	–	12	–	–	25
2.	*Candida albicans* MTCC 3017	30	30	15	14	–	27	19	18	11	–	18	–	–	–	–	25
3.	*Candida tropicalis* MTCC 184	14	11	–	–	–	13	–	18	–	–	21	–	–	–	–	20
4.	*Cryptococcus terreus* MTCC 1716	25	25	–	–	–	19	–	15	–	–	16	–	–	–	–	12
5.	*Saccharomyces cerevisiae* MTCC 170	21	23	–	–	–	25	28	20	–	–	23	–	11	11	–	20
6.	*Kluyveromyces lactis* MTCC32	27	24	–	–	21	21	31	20	–	–	29	–	13	13	–	12
8.	*Penicillium chrysogenum* MTCC 2725	19	11	–	–	–	–	–	–	–	–	20	–	–	–	13	–
9.	*Aspergillus niger* MTCC 1344	–	12	12	–	–	12	13	–	–	–	14	–	–	–	15	–
11.	*Trichophyton rubrum* MTCC 296	–	–	–	–	18	16	14	12	–	–	20	–	–	13	12	32
12.	*Beauveria bassiana* MTCC 4564	16	20	20	11	–	–	19	–	–	–	–	–	–	–	–	–

*(−), not active; PC, positive control; AmB, Amphotericin B and well diameter = 10 mm*.

### Characterization of the strains

The cultural characteristics, such as microbial growth along with its pattern and pigment formation were studied on International *Streptomyces* Project (ISP) media and their results are summarized in Table 3. All the selected actinomycetes isolates showed moderate to heavy growth on nitrate agar, urease agar and inorganic salt starch agar medium, which suggests their capability of breaking down the above complexes. Heavy growth and color change (from green to blue) of Simmons' Citrate Agar (SCA) medium provide the evidence of citrate utilizing ability of the actinomycetes strains YE21, YE22, and YE23. Likewise, melanin formation was demonstrated by the presence of brown to black patches on inorganic salt starch agar (ISP4) and YMG (yeast extract 4 g, malt extract 10 g, glucose 4 g, agar 20 g, water 1 L) medium as a result of the growth of the active strains. This was observed in almost all the selected strains.

**Table 3:** **Cultural and Biochemical characteristics of the producer strains**.

		**Producer strains**												
**Characteristics**		**H1**	**N1**	**S1**	**K1**	**V1**	**ZE 18**	**ZE 19**	**YE 21**	**YE 22**	**YE 23**	**ZA 25**	**ZA 26**	**RS25**
Growth on	ISP2	2+	+	2+	2+	2+	+	2+	3+	3+	2+	3+	–	3+
	ISP3	2+	+	2+	2+	2+	2+	2+	2+	3+	3+	–	2+	2+
	ISP4	3+	2+	2+	3+	3+	2+	3+	+	2+	3+	–	3+	3+
	ISP5	2+	2+	+	2+	+	+	2+	+	+	2+	–	2+	2+
	ISP6	3+	2+	2+	3+	2+	+	+	+	2+	2+	+	+	2+
	ISP7	+	2+	+	+	+	+	3+	2+	3+	2+	+	–	+
Utilization of	Citrate	–	–	–	+	+	–	–	2+	2+	2+	–	–	–
	Asparagine	3+	2+	+	–	2+	–	–	+	+	+	+	–	–
	Oat meal	2+	+	2+	–	2+	2+	2+	2+	3+	3+	–	2+	2+
	Tributyrin	2+	2+	2+	2+	3+	+	–	–	–	+	–	+	+
Enzyme production	Urease	–	–	−	−	−	+	−	2+	−	+	−	−	+
	Amylase	3+	2+	2+	2+	3+	2+	3+	+	2+	3+	−	3+	3+
	Tyrosinase	3+	2+	−	+	+	+	3+	2+	3+	2+	+	−	+
	Chitinase	3+	2+	2+	2+	+	−	−	3+	+	+	−	−	+
	Heparinase	−	−	−	−	3+	−	−	−	−	−	−	−	−
	Protease	2+	2+	2+	2+	2+	+	2+	3+	3+	2+	3+	+	3+
	Catalase	2+	2+	2+	2+	2+	+	+	2+	2+	3+	2+	+	+
pH tolerance	5	3+	3+	2+	3+	3+	3+	3+	3+	3+	2+	2+	2+	3+
	7	3+	2+	3+	3+	3+	+	2+	3+	3+	3+	+	+	3+
	9	2+	3+	+	3+	3+	+	+	3+	3+	2+	2+	+	3+
	11	−	2+	+	2+	3+	+	+	3+	3+	2+	+	+	2+
NaCl tolerance	1%	3+	3+	3+	3+	3+	+	3+	3+	3+	2+	2+	+	3+
	3%	+	2+	2+	3+	3+	−	+	3+	3+	3+	−	+	+
	5%	−	−	+	2+	2+	−	−	3+	3+	2+	−	−	+
	7%	−	−	+	+	+	−	−	+	3+	+	−	−	−
Growth at	28°C	3+	2+	3+	3+	2+	+	2+	3+	+	3+	+	+	3+
	37°C	3+	3+	2+	3+	3+	2+	2+	3+	+	3+	+	−	3+
	47°C	−	−	−	−	+	−	−	−	−	−	−	−	−

*(−), No growth; (+1), Poor growth; (+2), Moderate growth; (+3), Heavy growth*.

All the strains were capable of reducing nitrate salts but they demonstrated some differences against the decomposition of starch and urea. The production of amylase, urease and protease enzymes by the actinomycetes strains was endorsed by their growth on ISP4, urease agar and ISP3 mediums. Except the actinomycetes strains ZA25, ZA26, and YE22, the most favorable temperature range for the growth of strains was 27–37°C. Interestingly, none of the selected strains were able to grow at 50°C or above. All the selected actinomycetes strains possessed the ability to tolerate 3% NaCl in the medium with the exception of strains ZE18 and ZA25.

### Hierarchical cluster analysis

**Figure 3:**
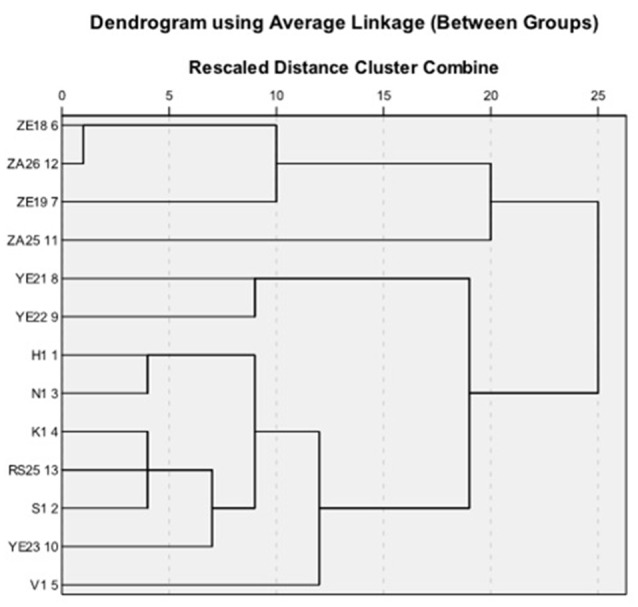
**Phylogenetic tree showing evolutionary relationship of the soil isolates**.

Strains found to be active in primary and secondary screening were subjected to hierarchical cluster analysis based on 49 macroscopic, biochemical and physiological characters. The dendrograms based on the average linkage between the groups were generated by PASW Statistics (formerly SPSS Statistics) Version 18 software. Two broad clusters were generated (Figure 3). The first cluster contained four (ZE18, ZE19, ZA25, and ZA26) and the second cluster contained nine actinomycetes (YE23, RS25, H1, V1, N1, S1, YE22, YE21, and K1) isolates. The first cluster was further divided into two sub cluster sub-clad 1.1 and 1.2. The sub-clad 1.1 leaded ZA25 alone whereas sub-clad 1.2 further divided into 1.2.1 and 1.2.2 and so on, and ultimately formed a branched group of three isolates (ZE18, ZE19, and ZA26). Similarly, the second cluster was divided into sub-clad 2.1 and 2.2. The sub-clad 2.1 was further divided into 2.1.1 and 2.1.2 (YE21 and YE2,), whereas sub-clad 2.2 was further divided into 2.2.1 and 2.2.2 and so on, and ultimately formed a branched group of seven isolates (YE23, RS25, H1, V1, N1, S1, and K1).

Based upon the potential antimicrobial activities shown in the primary and secondary screenings, five out of thirteen strains were finally selected for further characterization using 16S rRNA homology studies. The generated dendrogram showed that all the five strains (H1, V1, S1, RS25, and ZA25) were evolutionary far away from each other and displayed varying biochemical characteristics along with excellent antimicrobial activity. The diversity was maintained when we compared the antimicrobial activity data and the UV spectra of the metabolites. Strains, ZA25 and H1 belong to the main cluster I and II, respectively, found to produce both antibacterial and antifungal compounds having different UV absorbance. Similarly, H1, V1, and S1 produce antibacterial compounds and belong to the same cluster II; but their sub-clad were different. Variations in the UV absorbance of the metabolites supported the positional difference of the strains in the dendrogram. The 16S rRNA study for the strains H1, V1, and S1 were performed at Microbial Type Culture Collection (MTCC), Chandigarh, Punjab (India), and identified and submitted to MTCC as - *Streptomyces xanthophaeus* MTCC 11938, *Streptomyces variabilis* MTCC 12266, and *Streptomyces xanthochromogenes* MTCC 11937, respectively. Whereas, the 16S rRNA homology sequence study for the strain RS25 and ZA25 was done at Genomebio Technologies Pvt. Ltd., Pune, Maharashtra (India) and identified and submitted to National Collection of Industrial Microorganisms (NCIM), CSIR-NCL, Pune, Maharashtra (India) as *Streptomyces levis* EU 124569 and *Streptomyces* sp. NCIM 5500, respectively.

### Partial chemical characterization of the active compounds

The bioactive compounds were purified from the selected actinomycetes strains using silica column, with methanol:chloroform gradient as an eluting solvent system. The purified fractions were partially characterized by observing under UV absorbance (λ_max_) and their absorption pattern to gather some preliminary information regarding the structure of the compound(s) (Table 4). Absorbance within UV range confirmed the presence of “*unsaturation*” in all the bioactive compounds. All the active metabolites except from the strain RS25 contained either carboxyl or peptide moiety in their structure. Absorbance band near 253 nm indicated the presence of benzene moiety in H1F2 (antifungal; AF), H1F1 (antibacterial; AB) and ZA25 AF strains. Among antifungal compounds, absence of conjugated absorption suggested non-polyene nature of H1F1 and ZA25 AF. The presence of carboxyl or peptide moieties was further confirmed by IR spectra of the compounds. Hydroxyl and conjugated carbonyl functional moiety in the compound were confirmed by the presence of absorption bands at 3425 and 1648 cm^−1^, respectively. The absorption bands detected at 3020 and 2927 cm^−1^ correspond to aromatic C-H and alkyl C-H stretching, respectively, and suggested the presence of aromatic ring in the antibacterial compound isolated from ZA25 and absence in the compound isolated from V1. Further, presence of aromatic ring was verified by the absorption bands at 1602 and 1504 cm^−1^ attributed to C = C ring stretching.

**Table 4:** **IR spectra of bioactive compounds isolated from the selected actinomycetes strains**.

**Strains**	**Identification**	**Nature of purified compound**	**UV absorbance**	**IR frequency**
H1F1	*Streptomyces xanthophaeus*	Antibacterial	201, 260	3409, 3019, 2400, 1658, 1402, 1384, 1215, 1053, 948, 758, 668
ZA25F1	*Streptomyces* sp.	Antibacterial	264, 495	3411, 3019, 2400, 1647, 1403, 1070, 928, 669
V1	*Streptomyces variabilis*	Antibacterial	210	3412, 2928, 1646, 1215, 1070,
RS25	*Streptomyces levis*	Antibacterial	240-250 and 332	3425 and 1648, 3020 and 2927, 1602 and 1504 cm^−1^
S1	*Streptomyces xanthochromogenes*	Antifungal	205, 490	3411, 3020, 1646, 1403, 1385, 1216, 1069, 769
H1F2	*Streptomyces xanthophaeus*	Antifungal	201, 265	3850, 3431, 3020, 2399, 2110, 1637, 1403, 1385, 1017, 669
ZA25F2	*Streptomyces* sp.	Antifungal	205, 248, 263,	3850, 3366, 2945, 2833, 2518, 2042, 1638, 1449, 1218, 1113, 757, 666

## Discussion

During the screening of rare actinomycetes, fast growing bacterial colonies inhibits the colonization of actinomycetes on the isolation medium, hence in order to isolate actinomycetes, the growth of these bacteria should be inhibited. Pre-treatment of the soil samples reduced the growth of ubiquitous microbial species, thereby facilitated the recovery of less-abundant microorganisms. The spores of actinomycetes and fungi generally resist desiccation and show slightly higher resistance toward wet or dry heat than other microbes (Hopwood and Wright, 1973). Pre-treatment of the soil suspension with 1.5% phenol (30°C for 30 min) lowered the number of bacteria, fungi, and other common actinomycetes by denaturing their proteins or by disrupting their cell membrane, however phenol-resistant actinomycetes were less affected during this process (Hayakawa et al., 1991). Earlier, Hayakawa et al. (1991) and Kim et al. (1995) reported the use of above mentioned pre-treatment of the soil samples for the isolation of actinomycetes, and they reported similar results, when the soil samples were pre-treated.

A total of 15 isolated strains were subjected to antimicrobial screening and it was found that most of the isolates were active against gram positive bacteria. This was majorly attributed to the presence of lipopolysaccharide (LPS), a major structural unit of gram negative bacterial cell wall. LPS is hydrophobic in nature and makes the cell wall impermeable to lipophilic solutes, whereas in absence of LPS in the cell wall of gram positive bacteria, they become susceptible to the metabolites (Kim et al., 1994).

Out of 15 strains, thirteen showed strong antimicrobial activity and were selected for detailed microbial characterization studies. Melanin formation was also observed in almost all the selected strains, which is a main diagnostic feature of *Streptomyces* sp. (Singh et al., 2009). Further, five strains were characterized by using nucleotide sequencing and designated as H1: *S*. *xanthophaeus* MTCC 11938, V1: *S*. *variabilis* MTCC 12266, S1: *S*. *xanthochromogenes* MTCC 11937, RS25: *S*. *levis* EU124569, and ZA 25: *Streptomyces* sp. NCIM 5500. Evolutionary distance of the above five strains (H1, V1, S1, RS25, and ZA 25) was also analyzed by measuring their positions in the dendrogram. Based upon the diversity achieved by the morphological, biochemical, and the phylogenetic characteristics in the selected strains, we can speculate the possibility of involvement/role of diverse bioactive compounds from these strains, responsible for their broad-spectrum antimicrobial properties.

During the purification of the metabolites from ultra violet spectra, maximum absorbance between 205 and 216 nm suggested the presence of carboxyl or peptide moiety in the structure of all the active metabolites except from the strain RS25 (Singh et al., 2009; Kang et al., 2010). However, absorbance at 240–250 and at 332 nm predicted the presence of chromone like nucleus in strain RS25 (Griffiths and Ellis, 1972).

Pertinent literature about the producer strains were searched using SciFinder software program and it was found that *S. xanthophaeus* is known to produce diverse array of metabolites including Postproline endopeptidase, Benarthin, β-galactosidase-inhibiting isoflavonoids and geomycins (Brockmann and Musso, 1954; Hazato et al., 1979; Aoyagi et al., 1992; Shibamoto et al., 1993). *S*. *xanthochromogenes* are also known to produce diverse array of chemical compounds such as Diastereoisomeric I-Na, Nitropeptin, xanthicin (I), Pravastatin, reductiomycin, alkaloid AM-6201 (Arishima et al., 1956; Onda et al., 1982; Ohba et al., 1987; Otake et al., 1988; Cho et al., 1993; Zhang et al., 2008). Eleven compounds having same molecular formula have been reported from *S. levis*. Those were oleandomycin, 2-piperidinone and derivatives of either erythromycin or tylonolide. *S. variabilis* is reported for the production of L-Glutaminase, imunosuppresive, clavulanic acid, glycoside antibiotic and 1-hydroxy-1-norresistomycin (Abd-Alla et al., 2013, 2016; Marques et al., 2014; Ramalingam and Rajaram, 2016).

To the best of our knowledge, this is very first time we are reporting the antifungal activity of *S. xanthophaeus* and *S*. *xanthochromogenes*. Similarly, the compounds reported from *S. levis* didn't show the presence of chromone like structure. Also, the antifungal compound purified from *S*. *xanthochromogenes* was confirmed as chitinase by performing enzyme assay.

In conclusion, the results of morphological and biochemical characterization and the nature of compounds produced by the microbes established the diversity among the member of actinomycetes. The antimicrobial activities achieved in this study indicate that the isolated strains of *Streptomyces* sp. from different geographical niches of India have potential to produce diverse array of antimicrobial compounds that can be useful for many great applications and must be explored extensively. Currently, our research group and collaborators are actively involved in the chemical characterization of the active compounds identified in this study. In addition, *in silico* predictive studies for target prediction are under progress with our collaborator group. Hopefully, in our successive publication we would be reporting the complete information regarding the chemical characteristics of the “*actives*” identified in this study and their targets predicted through *in silico* binding studies and their experimental validation.

## Author contributions

Conceived and designed the study and experiments: VS, SH, HS, JV, KV, RS, AJ, CT. Performed the experiments: VS, HS, JV, KV, RS. Analyzed the data: VS, SH, AJ. Contributed reagents/materials/analysis tools: VS, SH, AJ, CT. Wrote the paper: VS, SH, AJ, CT. All authors reviewed the manuscript.

## Funding

The financial support for this study was available from the Department of Science and Technology, Ministry of Science and Technology, Government of India, New Delhi, under Fast Track Fellowship awarded to VS (Grant No. SR/FT/LS-190/2009).

### Conflict of interest statement

The authors declare that the research was conducted in the absence of any commercial or financial relationships that could be construed as a potential conflict of interest. The reviewer PCV declared a shared affiliation, though no other collaboration, with the authors to the handling Editor, who ensured that the process nevertheless met the standards of a fair and objective review.

## References

[B1] Abd-Alla M. H. El-Sayed E. S. A. Rasmey A. H. M. (2013). Biosynthesis of L-glutaminase by *Streptomyces variabilis* ASU319 isolated from rhizosphere of triticum vulgaris. Univ. J. Microbiol. Res. 1, 27–35. 10.13189/ujmr.2013.010301

[B2] Abd-Alla M. H. Rasmey A. H. M. El-Sayed E. S. A. El-Kady I. A. Yassin I. M. (2016). Biosynthesis of anti-inflammatory immunosuppressive metabolite by *Streptomyces variabilis* ASU319. Eur. J. Biol. Res. 6, 152–169. Available online at: http://www.journals.tmkarpinski.com/index.php/ejbr/article/view/455

[B3] Aoyagi T. Hatsu M. Kojima F. Hayashi C. Hamada M. Takeuchi T. (1992). Benarthin: a new inhibitor of pyroglutamyl peptidase. i taxonomy, fermentation isolation and biological activities. J. Antibiot. 45, 1079–1083. 10.7164/antibiotics.45.1079 1355471

[B4] Arishima M. Sakamoto J. Sato T. (1956). An antibiotic *Streptomyces* no. 689 strain. I. Taxonomic studies. Nippon Nogei Kagaku Kaishi 30, 469–471. 10.1271/nogeikagaku1924.30.8_469

[B5] Berdy J. (2005). Bioactive microbial metabolites, a personal view. J. Antibiot. 58, 1–26. 10.1038/ja.2005.1 15813176

[B6] Brockmann H. Musso H. (1954). Antibiotics from actinomycetes. XXIX. Geomycin 2. Chem. Ber. 87, 1779–1799. 10.1002/cber.19540871128

[B7] Cho H. Beale J. M. Graff C. Mocek U. Nakagawa A. Omura S. (1993). Studies on the biosynthesis of the antibiotic reductiomycin in *Streptomyces xanthochromogenus*. J. Am. Chem. Soc. 115, 12296–12304. 10.1021/ja00079a009

[B8] Griffiths P. J. F. Ellis G. P. (1972). Benzopyrones—VI1: the ultraviolet absorption spectra of chromone and 2-substituted chromones. Spectrochim. Acta A Mol. Spectros. 28, 707–713. 10.1016/0584-8539(72)80039-4

[B9] Hayakawa M. (2008). Studies on the isolation and distribution of rare actinomycetes in soil. Actinomycetologica 22, 12–19. 10.3209/saj.SAJ220103

[B10] Hayakawa M. Sadakata T. Kajiura T. Nonomura H. (1991). New methods for the highly selective isolation of Micromonospora and Microbispora from soil. J. Fermen. Bioeng. 72, 320–326. 10.1016/0922-338X(91)90080-Z

[B11] Hazato T. Naganawa H. Kumagai M. Aoyagi T. Umezawa H. (1979). Beta-Galactosidase-inhibiting new isoflavonoids produced by actinomycetes. J. Antibiot. 32, 217–222. 10.7164/antibiotics.32.217 110760

[B12] Holt J. G. Krieg N. R. Sneath P. H. A. Staley J. T. Williams S. T. (1994). Bergey's Manual of Determinative Bacteriology, 9th Edn. Baltimore, MD; Philadelphia; Hong Kong; London; Munch; Sydney; Tokyo: William and Wilkins.

[B13] Hopwood D. A. Wright H. M. (1973). A plasmid of *Streptomyces* coelicolor carrying a chromosomal locus and its inter-specific transfer. J. Gen. Microbiol. 79, 331–342. 10.1099/00221287-79-2-331 4772093

[B14] Kang M. J. Strap J. L. Crawford D. L. (2010). Isolation and characterization of potent antifungal strains of the *Streptomyces* violaceusniger clade active against *Candida albicans*. J. Ind. Microbiol. Biotechnol. 37, 35–41. 10.1007/s10295-009-0641-9 19784681 PMC2797434

[B15] Kekuda P. T. R. Onkarappa R. Jayanna N. D. (2014). Characterization and antibacterial activity of a glycoside antibiotic from *Streptomyces variabilis* PO-178. Sci. Technol. Arts Res. J. 3, 116–121. 10.4314/star.v3i4.17

[B16] Kim C. Lee K. Kwon O. Park D. Shimazu A. (1995). Isolation of rare actinomycetes on various types of soil. Kor. J. Appl. Microbiol. Biotechnol. 23, 36–42.

[B17] Kim C. Lee K. Kwon O. Yoo I. Shimazu A. (1994). Selective isolation of actinomycetes by physical pretreatment of soil sample. Kor. J. Appl. Microbiol. Biotechnol. 22, 222–225.

[B18] Kurtböke D. I. Chen C. F. Williams S. T. (1992). Use of polyvalent phage for reduction of streptomycetes on soil dilution plates. J. Appl. Bacteriol. 72, 103–111. 10.1111/j.1365-2672.1992.tb01810.x 1556035

[B19] Madigan M. T. Martiko J. M. Parker J. (1997). Antibiotics: isolation and characterization, in Brock Biology of Microorganisms, 8th Edn., ed Madigan M. T. (New Jersey: Prentice-Hall International Inc.), 440–442.

[B20] Marques D. A. V. Santos-Ebinuma V. D. C. de Oliveira P. M. S. de Souza Lima G. M. Araújo J. M. Lima-Filho J. L.. (2014). Screening of wild type *Streptomyces* isolates able to overproduce clavulanic acid. Braz. J. Microbiol. 45, 919–928. 10.1590/S1517-83822014000300022 25477926 PMC4204977

[B21] Ohba K. Nakayama H. Furihata K. Shimazu A. Endo T. Seto H.. (1987). Nitropeptin, a new dipeptide antibiotic possessing a nitro group. J. Antibiot. 40, 709–713. 3610825 10.7164/antibiotics.40.709

[B22] Okami Y. Hotta K. (1988). Search and discovery of new antibiotics, in Actinomycetes in Biotechnology, eds Goodfellow M. Williams S. T. Mordarski M. (London: Academic Press), 37–67.

[B23] Onda M. Konda Y. Hinotozawa K. Omura S. (1982). The alkaloid AM-6201 from *Streptomyces* xanthochromogenus. Chem. Pharmaceut. Bull. 30, 1210–1214. 10.1248/cpb.30.1210

[B24] Otake N. Seto H. Nakayama H. Endo T. Oba K. Iwata M. (1988). Antibiotic 6257 manufacture with Streptomyces for *Pyricularia oryzae* infection control in rice. Jpn. Kokai Tokkyo Koho. JP 63126495 (Accessed May 30, 1988).

[B25] Praveen V. Tripathi D. Tripathi C. K. M. Bihari V. (2008). Nutritional regulation of actinomycin-D production by a new isolate of *Streptomyces* sindenensis using statistical methods. Indian J. Exp. Biol. 46, 138–144. 18335813

[B26] Ramalingam V. Rajaram R. (2016). Antioxidant activity of 1-hydroxy-1-norresistomycin derived from *Streptomyces variabilis* KP149559 and evaluation of its toxicity against zebra fish *Danio rerio*. RSC Adv. 6, 16615–16623. 10.1039/C5RA22558B

[B27] Shibamoto N. Terasawa T. Okamoto R. Shin T. Murao S. (1993). Novel postproline endopeptidase and its manufacture with Streptomyces species. Jpn. Kokai Tokkyo Koho. Patent No. JP 05244947, Indexing: Fermentation and Bioindustrial Chemistry (Section 16-4).

[B28] Shirling E. B. Gottlieb D. (1966). Methods for characterization of *Streptomyces* sp. Int. J. Syst. Bacteriol. 16, 313–340. 10.1099/00207713-16-3-313

[B29] Singh V. Tripathi C. K. M. (2011). Olivanic acid production in fed batch cultivation by Streptomyces olivaceus MTCC 6820. Res. J. Pharmaceut. Biol. Chem. Sci. 2, 726–731.

[B30] Singh V. Praveen V. Banga J. Tripathi C. K. M. (2009). Antimicrobial activities of microbial strains isolated from soil of stressed ecological niches of Eastern Uttar Pradesh, India. Indian J. Exp. Biol. 47, 298–303. 19382727

[B31] Takahashi Y. Omura S. (2003). Isolation of new actinomycete strains for the screening of new bioactive compounds. J. Gen. Appl. Microbiol. 49, 141–154. 10.2323/jgam.49.141 12949697

[B32] Tanaka Y. Omura S. (1990). Metabolism and products of actinomycetes. An introduction. Actinomycetolgica 4, 13–14. 10.3209/saj.4_13

[B33] Williams S. T. Goodfellow M. Alderson G. (1989). Bergey's manual of systematic bacteriology, in Genus Streptomyces Waksman and Henrici 1943, 339AL, Vol. 4, eds Williams S. T. Sharpe M. E. Holt J. G. (Baltimore, MD: Williams and Wilkins), 2452–2492

[B34] Wu R. Y. (1984). Studies on the Streptomyces SC4. II Taxonomic and biological characteristics of Streptomyces strain SC4. Bot. Bull. Acad. Sin. 25, 111–123.

[B35] Zhang L. Zhang J. Yang W. Bai G. (2008). Classification of Streptomyces strain Z314 and purification of its product pravastatin. Wei Sheng Wu Xue Bao 48, 33–37. 18338573

